# *Aceh Free Pasung*: Releasing the mentally ill from physical restraint

**DOI:** 10.1186/1752-4458-5-10

**Published:** 2011-05-14

**Authors:** Ibrahim Puteh, M Marthoenis, Harry Minas

**Affiliations:** 1 Department of Psychiatry, Syiah Kuala University, Darussalam, Banda Aceh, 23111, Indonesia; 2 Aceh Mental Hospital, Jl. Dr Syarif Thaib no 25, Banda Aceh, Indonesia; 3 STIkes Medika Nurul Islam, Sigli, Aceh, Indonesia; 4 Centre for International Mental Health, Melbourne School of Population Health, The University of Melbourne, Parkville, Victoria 3010, Australia; 5 International Technical Adviser to the Aceh Free Pasung Program, Government of Aceh, Indonesia

## Abstract

**Background:**

Physical restraint and confinement of the mentally ill (called *pasung *in Indonesia) is common in Aceh. In early 2010, the local government initiated a program called *Aceh Free Pasung *2010. The main goal of the program is to release the mentally ill in the province from restraint and to provide appropriate medical treatment and care. The aim of the paper is to report the findings of a preliminary investigation of the demographic and clinical characteristics of patients who have been admitted to the Banda Aceh Mental Hospital as part of the *Aceh Free Pasung *program.

**Methods:**

This is a cross-sectional descriptive study conducted at the Banda Aceh Mental Hospital, where people who had been restrained or confined in the community are being admitted for psychiatric treatment and, where necessary, physical rehabilitation, as part of the *Aceh Free Pasung *program.

**Results:**

Fifty-nine of former ex-*pasung *patients were examined. The majority (88.1%) of the patients were male, aged 18 to 68 years. The duration of *pasung *varied from a few days to 20 years, with a mean duration of 4.0 years. The reasons for applying *pasung *are many, with concerns about dangerousness being most common. The great majority (89.8%) had a diagnosis of schizophrenia.

**Discussion:**

The development of a community mental health system and the introduction of a health insurance system in Aceh (together with the national health insurance scheme for the poor) has enabled access to free hospital treatment for people with severe mental disorders, including those who have been in *pasung*. The demographic and clinical characteristics of this group of ex-*pasung *patients are broadly similar to those reported in previous studies.

**Conclusions:**

The Aceh Free *Pasung *program is an important mental health and human rights initiative that can serve to inform similar efforts in other parts of Indonesia and other low and middle-income countries where restraint and confinement of the mentally ill is receiving insufficient attention.

## Background

The prevalence of mental illness in Aceh (14.1% of the population of 4.2 million) is higher than the mean prevalence for Indonesia (11.6%). The prevalence of severe mental disorders in Aceh is 1.8% [1]. Multiple factors are thought to contribute to this observation, including poverty, the devastation caused by the 2004 tsunami [2,3] and particularly high levels of mental disorder in the areas most affected by the long-running military conflict [4].

While the need for mental health services is great, development of community mental health services only commenced in the post-tsunami period [2,5], and there is still much to do to achieve population coverage and quality of services. Faced with the problem of looking after a severely mentally ill family member, concerns about risk to the mentally ill member or to others, and inaccessible, unaffordable, ineffective psychiatric treatment services, many families (and village community leaders) have little option but to physically restrain the ill family member [6]. This practice, known in Indonesia as *pasung *[7-9], is now known to be widespread. The forms of restraint - in Indonesia and elsewhere - include securing ankles in wooden stocks, chaining and tying by rope to immovable objects (e.g. a building or a tree), locking in a confined space such as a cage or a box, and often a combination of confinement and restraint [10-13]. Such restraint and confinement may be brief and intermittent or it may persist for decades [14]. A frequent reason given in the past for applying *pasung *(or re-applying *pasung *after treatment has either failed or could not be sustained) has been inability to meet the necessary costs [6,8]. Despite the fact that such practices constitute a severe form of abuse of the human rights of persons with mental illness they have attracted little sustained attention from policy-makers, human rights activists and professional groups [8].

As part of a broad program of community mental health service development, in which Aceh has been the leader in Indonesia, the government of Aceh has turned its attention to protecting the human rights of people with *severe *mental illness, particularly those who have been restrained or confined in the community. When the Deputy Governor of Aceh, Muhammad Nazar, announced in March 2009 the intent of the government to establish the *Aceh Free Pasung *program there were 110 known cases of *pasung *in Aceh. The program includes the building of a special ward in the psychiatric hospital in Banda Aceh and the assignment of hospital staff to a special team that releases patients from *pasung *and brings them to the hospital for treatment in the new ward. In February 2010, after the building of the ward for *pasung *patients at the Banda Aceh Mental Hospital, the Governor of Aceh, Irwandi Yusuf, formally committed the government of Aceh to "remove the chains from the mentally ill" [15]. At this time, with further active case finding, there were at least 200 known cases of *pasung *in the province. The governor removed the chains from a 45 year old man who had been chained for 15 years. "We hope that no more mentally ill patients will be chained up or put in stocks in family backyards. The local government will take them to the mental hospital for proper treatment. It's a humanitarian mission because those with mental illnesses deserve proper treatment, just like the rest of us" [15].

The aim of this paper is to report the findings of a preliminary investigation of the demographic and clinical characteristics of patients who have been admitted to the Banda Aceh Mental Hospital as part of the *Aceh Free Pasung *program.

## Methods

This cross-sectional descriptive study was carried out in Banda Aceh Mental Hospital. Study participants were all patients who had been in *pasung *and were inpatients in the hospital during August 2010.

Since the start of the *Aceh Free Pasung *project 93 ex-*pasung *patients have been hospitalized for treatment. Thirty four patients had been discharged prior to the commencement of this study and 59 (63.4% of all ex-*pasung *patients admitted) were inpatients during the data collection period. Data about the patients discharged prior to the commencement of the study were not available to the researchers. The data collected were derived from the hospital medical records, and interviews with patients, ward nurses, and family members who were available at the hospital for interview or could be contacted by telephone.

Clinical diagnostic interviews and general medical examinations were carried out by a psychiatrist (IP).

Permission to carry out the study was granted by the Director of Banda Aceh Mental Hospital.

## Results

The main findings are summarised in Table 1.

**Table 1:** Demographic, *pasung *and clinical features

	Number/years	Percent/range
Sex		
Male	52	88.1%
Female	7	11.9%

Age: mean (range) years	34.9 years	18-68 years

Education		
No formal education	3	5.1%
Attended primary school	29	49.2%
Attended junior high school	15	25.4%
Attended senior high school	12	20.3%

*Pasung *initiated by family	51	86.4%

Violence/dangerousness given as main reason for *pasung *	47	79.7%

Duration of *pasung: *mean (range)	4.0 years	<1-20 years

Duration of illness: mean (range)	11.3 years	1-38 years

Mode of referral to hospital		
Brought to hospital by family	21	35.6%
Referred by community mental health nurses inprimary health centre	17	28.8%
Hospital Free Aceh *Pasung *team	20	33.9%
Referred by police	1	1.7%

Diagnosis of schizophrenia		89.8%
Schizophrenia, paranoid	53	89.8%
Schizophrenia, undifferentiated	3	5.1%
Schizoaffective Disorder	1	1.7%
Bipolar Affective Disorder	1	1.7%
Cannabis-induced Psychotic Disorder	1	1.7%

Previous psychiatric treatment	47	79.7%

Payment of hospital treatment costs		
Jaminan Kesehatan Aceh/Aceh Health Insurance	24	40.7%
Jamkesmas (Jaminan Kesehatan Masyarakat/Community Health Security	34	57.6%
"Poor identification letter"	1	1.7%

Fifty of the 59 patients (88.1%) who participated in the study were male, the mean age was 34.8 years, with a range of 18-68 years. Thirty two (54.3%) of the patients had not had education beyond primary school.

The main forms of physical restraint and confinement of persons with mental illness in Aceh are chaining, securing a leg or arm in wooden stocks (called *noh*), confinement in a locked small room or other space, or a combination of methods [6,8]. Such restraint often causes physical injury. The most common method for this sample of patients was using a metal chain, followed by the use of wooden stocks. Thirty seven (62.7%) of the patients had been restrained with a combination of methods. In a small number of patients *pasung *had been applied intermittently, only during periods when the mentally ill person was considered by the family or community to be at risk of harm or was aggressive and potentially dangerous.

The reasons for restraint were multiple. In the case of 47 patients (79.7%) the main concern was aggressive behaviour. In the remaining 12 (20.3%) cases there had been concern about the mentally ill person's safety and well-being because of wandering, and for a small number variety of "special reasons" was given. For example, there were some cases where patients had been restrained soon after the *Aceh Free Pasung *program was launched, in the hope that "someone from the government will come, release and take him to the hospital for treatment without having to pay any money". The decision to apply *pasung *had in most cases (86.4%) been made by the ill person's family. In the remainder of the cases (13.6%) the decision to apply *pasung *had been made by community leaders.

The location in which persons with mental illness were restrained varied. Some patients were restrained in a room of a house, in a small shelter behind the main house, under the traditional Acehnese stage house or under a tree in the yard or garden. One person was restrained in a garden two kilometres from the closest village, under a tree with his legs in stocks. Another person had her leg chained to a tree in a garden. She was able to walk, cook, and do some limited activities, and had some cooking equipment around her, but her food supply was limited.

The duration of *pasung *varied from only few days to 20 years, with a mean duration of 4.0 years. Almost half of the patients (27: 45.8%) had been in *pasung *for less than one year. Patients with longer durations of *pasung *were less likely to have received previous psychiatric treatment, and those with shorter durations of *pasung *had generally received treatment on at least one previous occasion.

Twenty one (35.6%) of the admitted ex-*pasung *patients had significant atrophy of muscles in their leg or arm at the time they were released from *pasung *and admitted to hospital. Nearly all those with lower extremity atrophy had difficulty walking, approximately half could not walk at all, and all required physical therapies during their hospitalisation.

The diagnoses recorded in the medical files were validated by a psychiatrist (IP). Fifty three of the 59 patients (89.8%) had a diagnosis of schizophrenia. The mean duration of illness was 11.3 years, with a range from a few days to 38 years. The majority of the patients (47: 79.7%) had been previously admitted for treatment to Banda Aceh Mental Hospital, with between one and eight previous admissions. Only twenty two of the 59 patients (20.3%) of the patients had had no previous treatment. Many of the respondents indicated that in addition to previous hospital treatment they had also sought treatment from traditional or religious healers. Approximately one quarter of the patients who had never been hospitalized before the launch of the new health insurance arrangements gave inability to pay for hospital services as the reason they had not previously sought treatment.

Roughly equal numbers of patients had been sent to the hospital by their family (21: 35.6%), referred by a community mental health nurse from a primary care health centre (17: 28.8%), and released from *pasung *and taken to the hospital by hospital staff as part of the *free pasung *program (20: 33.9%). Only one case (1.7%) had been referred to the hospital by police.

The cost of medical treatment for the majority of the patients was covered by social health insurance. Virtually all of the patients were covered by the two main forms of health insurance, JKA (Jaminan Kesehatan Aceh/Aceh Health Insurance) (24: 40.7%) and by Jamkesmas (Jaminan Kesehatan Masyarakat/Community Health Security) (34: 57.6%). None of the patients had any out-of-pocket expenditure as a result of the inpatient treatment, apart from the cost of transport to the hospital. For approximately half of the patients the costs were paid by the new Health Insurance Aceh scheme and for the other half by Jamkesmas, the national insurance scheme that aims to guarantee access to health services for Indonesia's poor.

## Discussion

In this paper we report the characteristics of patients admitted to Banda Aceh Mental Hospital as part of the *Aceh Free Pasung *program, an innovative government program intended to protect the human rights of people with severe mental illness and to eliminate the practice of *pasung *from the province of Aceh. Almost 90% of the ex-*pasung *patients admitted to hospital were male, almost 90% had a diagnosis of schizophrenia, and in 80% the main reason for applying *pasung *was an actual history or concern about violence and dangerousness. This combination of psychotic disorder in a male and concerns about dangerousness is a common constellation of features for people who have been in *pasung *in Aceh [6].

In June 2010 the government of Aceh launched the *Sistem Kesehatan Aceh *(Health System Aceh), which includes a local health insurance system called *Jaminan Kesehatan Aceh *(Health Insurance Aceh) that provides full and free access to health care services to the population of the province. The health insurance premium is paid by government. This is a substantial advance in equity and financial accessibility of health services in Aceh, and is a practical example of steps to achieve one of the key goals of the WHO Mental Health Global Action Program, "to enhance mental health services and to reduce disease burden" [16]. A continuing impediment to access to health care, particularly for poor families living in remote areas, is the cost of transport, which is not covered by health and social insurance.

The introduction of Health Insurance Aceh has resulted in changes in the community's attitude toward health care seeking and large increases in the number of people seeking health care at the provincial general hospital [17]. and is also likely to have increased demand for mental health services. Providing financially accessible health care for the mentally ill is an essential element in increasing mental health system coverage and improving population mental health in low and middle-income countries, particularly for the poor.

The fact that two-thirds of the patients were sent by their family or referred by the community mental health nurses from primary health care centres would suggest that there is a greater readiness both in the general population and among primary health care staff to expect that effective treatment is available. That the rest were identified by the hospital staff as part of their *free pasung *program activities is a clear indication that there is now greater attention to active case finding of persons in *pasung *than was the case in the past.

The print and electronic media in Aceh (and in Indonesia and internationally [18]) have played a very positive role in bringing *pasung *(and other human rights abuses [19]) to the attention of the general public, and health system bureaucrats and politicians. The media have also been active in informing the general public about the new health system and health insurance arrangements that have made free medical treatment available to the population of the province. This illustrates the importance of active collaboration with the media (as well as other key stakeholders) in improving human rights protections for the mentally ill, and in celebrating progress towards the achievement of such human rights goals [20].

For the program to achieve its goal of eliminating pasung from Aceh several objectives will need to be accomplished. There needs to be active and continuous case finding. Effective clinical treatment has to be available and delivered in a timely and affordable manner. The main challenge is after discharge from the Banda Aceh Mental Hospital. If people with severe mental disorder are to remain free of restraint they must have affordable access to continuous treatment as long as it is required, there must be sufficient support for social and economic engagement, concerns about dangerousness must be effectively allayed, and there must be adequate support for families and communities. In short, the elimination of the practice of *pasung *from Aceh will require the development of adequate and accessible community-based mental health and social support services, and education of the population about mental health and mental health services.

## Conclusion

It is not possible at this stage to make any judgment concerning the effectiveness of this program. This will be the subject of program evaluation efforts and further research. However, it is clear that an important beginning has been made. The *Aceh Free Pasung *program is an initiative of great importance, for Aceh, Indonesia and other low and middle-income countries where restraint and confinement of persons with mental illness continues. It is the first such government program of which we are aware in any of the countries in which restraint and confinement of mentally ill persons is known to occur. The efforts of the government of Aceh have now been joined by the Indonesian Ministry of Health which has declared that *pasung *will be eliminated from the whole of Indonesia by 2014 [21].

## Competing interests

The authors declare that they have no competing interests.

## Authors' contributions

IP conceived the study. IP and M carried out the data collection and initial data analysis and wrote a first draft of the manuscript. HM substantially revised and extended the manuscript and wrote the final version. All authors have seen and approved the final version of the manuscript.
